# The Essential Guide to Computer System Validation in the Pharmaceutical Industry

**DOI:** 10.7759/cureus.67555

**Published:** 2024-08-23

**Authors:** Jawahar Rohith Raja, Alekhya Kella, Damodharan Narayanasamy

**Affiliations:** 1 Department of Pharmaceutical Quality Assurance, SRM College of Pharmacy, SRM Institute of Science and Technology, Chengalpattu, IND

**Keywords:** qualification, v model, validation, gamp, computer system validation

## Abstract

This review aims to provide an essential guide to computer system validation (CSV) in the pharmaceutical industry. CSV is a process for ensuring that computer-based systems produce data and information that meets a set of pre-defined requirements. The pharmaceutical industry relies on data integrity to ensure reliable, accurate, and consistent information throughout the product lifecycle. Nowadays, since CSV plays an important role in the production and the final stages of a product, it is vital to ensure the reliability of the computer system, ensuring that it meets predefined requirements. The life cycle of CSV starts from the planning stage to the modification stage. Validation involves design, installation, operational, and performance qualifications (PQ), starting with a master plan and ending with periodic system reviews. Failure to validate the computer system leads to various regulatory compliances. Validating computer systems offers numerous benefits, including improved quality, reduced validation costs and time, and improved GMP compliance with 21 CFR part 11 regulations. Validating the computer system significantly affects how well, securely, and accurately products are made, ensuring they meet GxP requirements.

## Introduction and background

Quality is the most crucial requirement in all pharmaceutical and healthcare industries. We must produce every medication with the utmost care and quality. End-product testing alone cannot ensure quality; instead, we must closely monitor every crucial stage of the manufacturing process. In the pharmaceutical industry, it is crucial to properly validate and qualify all instruments and equipments. These steps are fundamental to a company’s commitment to quality assurance, making sure that everything works correctly and consistently, thus protecting product integrity and meeting regulatory requirements. The proper functioning of computer systems is important for maintaining quality in manufacturing. Two scientists named Byers and Loftus introduced the concept of validation to enhance pharmaceutical product quality. Validation is crucial for pharmaceutical and medical device production, as mandated by global regulations. Validating computer systems guarantees that these systems deliver information that is accurate and fulfills defined criteria [1]. An IT system is essential for pharmaceutical plants as it controls, supports, and documents processes. Validating computer and IT systems ensures that all applications function properly. Computer system validation (CSV) involves ensuring that computers, which control processes or gather analytical data, are functioning properly. This includes verifying all software and hardware that impact product quality, whether directly or indirectly [2]. The methods used to validate programmable logic controllers (PLCs) and personal computers (PCs) are quite similar to each other and align with general validation practices. In all cases, it is crucial for the end-user to define each specific requirement [1]. From start to finish, the validation process involves several steps: planning, defining requirements, coding, testing, installation, documenting everything, running the system, keeping an eye on its performance, and making adjustments as needed. The V-model facilitates structured testing, which is crucial for successful CSV. Validation relies on a series of documented checks and tests, like design qualification (DQ), installation qualification (IQ), operational qualification (OQ), and performance qualification (PQ), to confirm that the computer system works exactly as it was designed to [2].

## Review

The need for computer system validation

Computer software has become a significant tool for the pharmaceutical sector [3]. Pharmaceutical facilities necessitate precise procedures to guarantee top-notch final products. Validation is a methodical process that affirms the operation of a process within set parameters, guaranteeing uniform and reproducible outcomes within predetermined criteria. Pharmaceutical facilities must ensure real-time adherence to quality standards and regulations and compliance with current good manufacturing practice (cGMP) guidelines set by regulatory authorities. Validation serves as tangible proof that the process aligns with predetermined specifications [4]. Computer system software commonly undergoes design, development, and testing using software tools [5]. The pharmaceutical industry uses expensive raw materials, advanced processes, and state-of-the-art facilities and machinery to produce its final products. To maintain the integrity and quality of these products, it is essential to establish a robust system that meets company standards. Today, computerized systems are heavily involved in both the manufacturing and final stages of production. Ensuring these systems function correctly is crucial for consistently delivering high-quality products [6].

Regulatory requirements 

The FDA regulations outline the requirements for CSV, FDA is the agency responsible for protecting public health: The FDA 21 CFR part 820.70, FDA, 21 CFR part 11.10, FDA, 21 CFR part 11, European Union Annex 11, A Guidance Document about Software Validation from the FDA, GMP Directives, ISO 13485, and Good Automated Manufacturing Practice (GAMP) 5 [7,8].

Areas to be validated

**Figure 1:**
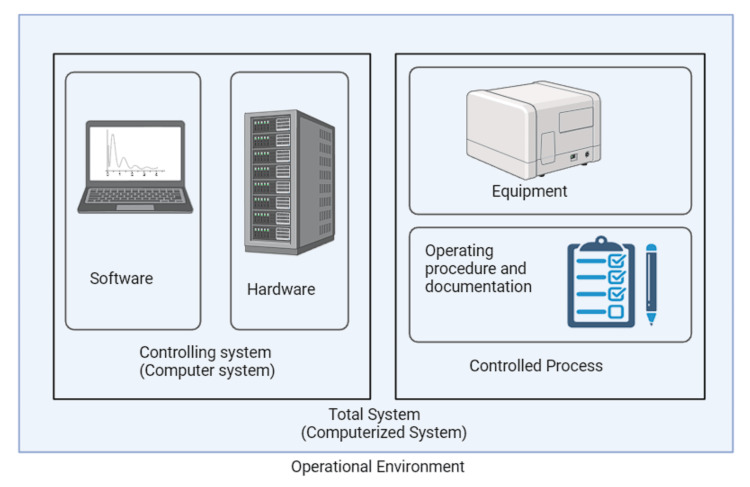
Computerized system Areas for validation and qualification of a computer system from "computer system validation: a review." Yogesh P, Kamlesh M, Mohini B, et al. (2015).

Computer systems include computer hardware, software, networks, accessories, cloud services, employees, and supporting documentation [2]. For instance, Figure 1 displays user manuals and standard operating procedures.

Good Automated Manufacturing Practice

Pharmaceutical sector manufacturers and computerized machinery users can refer to the GAMP as an array of standards. These guidelines provide guidance on risk management systems and contribute to computer system development. Additionally, GAMP is an International Society for Pharmaceutical Engineering (ISPE) technical subcommittee that addresses problems related to the product lifecycle. GAMP incorporates quality into every stage of the production process, instead of testing it in a single product batch. Therefore, GAMP encompasses all production features, including raw materials, equipment, training, and staff hygiene. GAMP's objective is to offer an affordable structure of standards to guarantee that computerized systems are efficient, suitable for their intended purpose, high-quality, and comply with relevant laws [9]. CSV's actions are based on GAMP 5's two classification categories: GxP impact, requirements for digital records and verification, DI, and a risk-based lifecycle plan [10].

GAMP 5 Categories

This breakdown focuses on the main categories. Table 1 and Table 2 delineate the categorization of items into two primary categories: software and hardware [8].

**Table 1:** Software types according to GAMP 5 Categories of GAMP 5 from "Computerized systems validation (CSV) in biopharmaceutical industries," Hesham AM. GAMP, Good Automated Manufacturing Practice; DCS, distributed control system; BMS, business management software; ERP, enterprise resource planning; SCADA, supervisory control and data acquisition; DS, design specification

Type	Explanation	Method of Validation	Example
Type 1 software for infrastructure	Layered software - programs for controlling the operating system.	Document the model number and confirm the proper installation by adhering to the approved installation procedures.	Some of the software, such as statistical packages, spreadsheets, and network monitoring tools.
Type 2 control software	Not anymore in use.		
Type 3 non-configured software	Runtime parameters can be entered and stored, but the software cannot be customized to fit the business process.	URS, or the abbreviated life cycle approach, is a risk-based method for evaluating suppliers. Note the version number, confirm the installation correctly, and run risk-based tests in accordance with the user's instructions. protocols in place to ensure compliance and appropriateness for the designed use.	Software examples such as laboratory software and PLC software and firmware-based applications.
Type 4 configured software	Software, often highly complex, that users can configure to meet the particular requirements of their business processes without altering the software code.	Life cycle approach: An approach to supplier assessment that is based on risk shows that the supplier has a sufficient quality management system and that some life cycle documentation (such as the DS) is retained solely by the supplier. Record the number and check the version to make sure the installation is correct. Utilizing risk-based testing, applications can be shown to function in the evaluated environment as intended. risk-based testing to show that the application functions within the business process as intended. protocols in place to ensure compliance and suitability for the intended use. protocols established for data management.	Here are some examples, such as SCADA, ERP, DCS, and BMS.

**Table 2:** Hardware types according to GAMP 5 Categories of GAMP 5 from "Computerized systems validation (CSV) in biopharmaceutical industries." Hesham AM. GAMP, Good Automated Manufacturing Practice; IQ, installation qualification;  DS, design specification

Type	Hardware type	Method of validation	Example
Type 1	Standard hardware type	It is recommended to document standard hardware components with their version number, make, and supplier information.	The programmable logic circuit and controller scanner
		The hardware data sheet contains information on the hardware.	
Type 2	Custom-built hardware type	Hardware needs to go through acceptance testing and have a DS.	Type 2 is a printed circuit board.
		Any hardware configuration ought to be specified in the IQ and verified in the design documentation.	

Life cycle model

**Figure 2:**
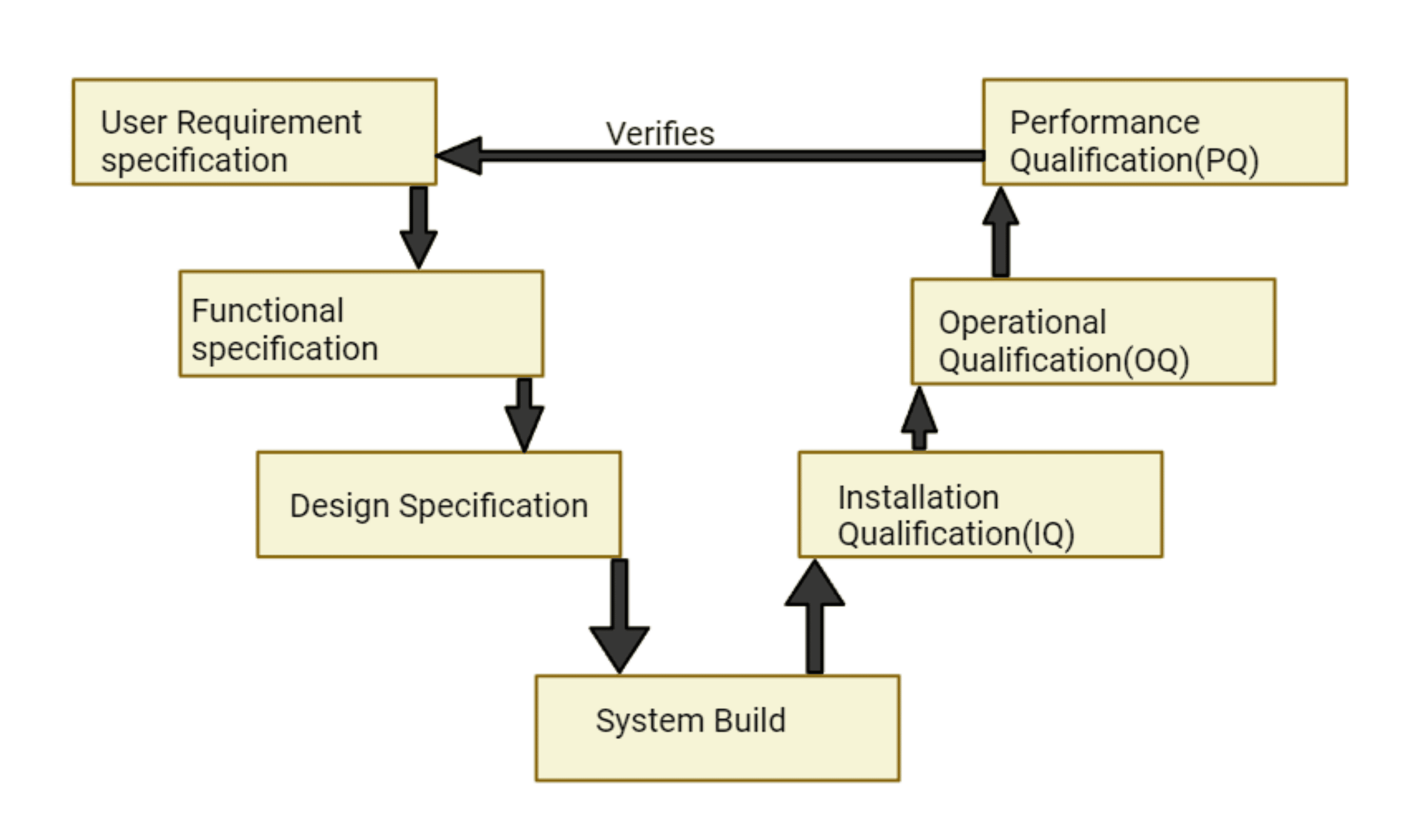
V-model of qualification

The goals of using a life cycle model for verification and validation align with those of a system architect. Controlling a system of defined elements, such as business or organizational functions, allows for efficient and cost-effective work processes and products [11]. Throughout the software development process, specific validation and verification duties transform into thorough inspections, leading to the development of crucial aspects of the software life cycle [12]. We develop software systems using a life cycle model. A computer validation procedure connects every stage of the software system development process. As a result, this model can represent every stage of the CSV process. This facilitates the development process's effective documentation [13]. This provides insight into the necessary specifications of the system, including structured development activities, execution, utilization, and the discontinuation of computer programming. The figure below illustrates the complete life cycle of a computer system. The four stages of the computer system life cycle, as outlined in GAMP 5 (Global Association of Pharmaceutical Industry) Pharmaceutical Engineering (ISPE, 2008), concentrate on the engineering aspects of pharmaceuticals. Validation for the computer system project involves planning, specifying, developing, verifying, and reporting phases. During the project phase, complete CSV using the V-model is required. IT and CSV commonly use the V-model (Figure 2) to mitigate quality risks associated with computer-aided systems [14].

This approach includes code creation and evaluation, functional specifications (FS), design specifications (DS), IQ, OQ, PQ, and user requirement specifications (URS). The above-mentioned method is excellent, provided software development is also part of the validation process. It does not, however, cover some crucial steps, such as vendor assessment. Additionally, it appears to be too complicated for genuine off-the-shelf commercial customization [15].

The process of validating a computer system

The system design phase is where computer systems are validated. It starts and proceeds through the whole SDLC. The following are the various phases of CSV [2].

Validation Master Plan

A master plan is the process of creating an outline for the validation of a whole computer system. Since this is the primary validation process, it covers every aspect of established systems, including software and hardware, as well as validated processes like risk reduction [16]. The validation master plan covers equipment with computerized control and data acquisition systems (SCADA and DAS) that affect GxP. The validation master plan establishes broad guidelines for a range of validation activities, carried out in accordance with the validation approach as outlined in GAMP 5 and CFR Part 11. It also defines the degree of the validation program. The Validation Management Program (VMP) ensures the efficient and seamless completion of validations throughout the entire organization, while also meeting excellence, company, and government standards [17].

User Requirement Specification

The user documents his thoughts about the computer system in this example, the SCADA (supervisory control and data acquisition system). Every requirement has its own unique identifier (e.g., URS-01) to ensure verification of the criteria in subsequent validation steps and to help prevent removing any of the planned functionalities during the computer system development procedure. A distinctive designation facilitates the control of whether every system feature has undergone testing [14]. Give the system supplier enough details for their functional design specification (FDS) so they can estimate the time, money, and resources needed to engineer and document the computer system within a validation life cycle [2].

Design Qualification

This qualification should show that the design complies with GMP. Design principles for equipment should align with the goals of GMP. It is important to review the mechanical drawings and design elements that the equipment's manufacturer has provided [18]. This is significant because the vendor will construct the computer system in accordance with the FS, software DS, and hardware DS. After reviewing the documents and comparing the URS with the FS, SDS, and HDS, the appointed responsible parties approve the created record. The formal term for it is design qualification or DQ [19].

Installation Qualification

Installation qualification, or IQ for short, is the process of verifying the installation procedures listed in the "Installation Guide" document and validating the provided software installed in the designated environment with the specified configurations. The most important stage is software IQ, and usually, many problems will arise during this phase. Variations in the environment can lead to multiple issues, as the development environment may not always provide a 100% real-time environment for verifying installation issues and there may have been some documentation issues during the process of recording the actual installation steps in the document, as they may not have precisely matched the production environment [1]. The computer system can proceed to OQ after issuing an acceptable and authorized IQ summary report [15].

Operational Qualification

The OQ test verifies both the system's functionality and the validity of the IQ protocol. It involves both static and dynamic testing, ensuring accurate testing and documentation of all continuous, sequential control and graphically displayed component activities [13]. "The stated collection of activities required to demonstrate that an instrument will function in accordance with its operational specification in the chosen environment" is known as OQ. It is important to underline the phrase "in the selected environment." Testing of the instrument hardware at the user's location is required because equipment properties may vary due to physical vibration during transit from the supplier to the user. The most commonly asked questions about OQ testing are what to examine, what are the minimum requirements for acceptance, and who will conduct the tests. "Users, or their qualified designees, should perform these tests to verify that the instrument meets supplier or user specifications in their own environment," states URS in response to all of the questions [4].

Performance Qualification

After successfully completing the installation and OQ activities, the next stage of the validation process confirms that the product or software satisfies the specified performance aspects under the expected load consistently and without creating any bottlenecks in the production environment. PQ's primary goal is to guarantee that software will operate as intended on the anticipated system. We perform PQ to ensure that the software consistently meets the required performance standards under various load conditions, mirroring real-world scenarios. Therefore, PQ will require some time to complete, as daily tests are necessary to monitor the software system's actions [1].

Validation Summary Report

A summary report will be created based on the results obtained in the qualifications test [17].

Periodic Review

Computerized systems ought to undergo regular assessments to ensure their continued validity and compliance with GMP [20]. We regularly check the integrity of the computer-based system's validation status [17]. Concurrently with the change control procedure, we typically review all relevant validation records to determine the potential extent of revalidation. Reviews can occur more or less regularly, typically once a year, depending on the application. In addition to these evaluations, one can use internal audits to ensure proper protocol use and record control for validation support [15].

Common FDA inspectional deficiencies preventable through robust CSV

Non-compliance Due to an Unvalidated Regulatory System

Inspectors frequently pay attention to validation steps and the corresponding records produced during these processes. Inspectors typically look for documented evidence to confirm that they conducted validation steps in accordance with the approved process when it comes to CSV. Maintaining incomplete documentation may lead to noncompliance. Furthermore, the records provided should demonstrate compliance with relevant regulatory requirements.

Lack of a Documented Process

Procedures are frequently among the first documents an inspector requests because they provide a pre-established, approved framework for carrying out duties in a regulated environment. Validation procedures should clearly identify the deliverables and provide a detailed description of the validation operations. A precise definition of the validation approach is necessary, accounting for pertinent requirements and detailing the methodology employed. Identifying process stakeholders and precisely defining their roles and duties is also crucial [4].

Written Process Not Followed

When performing system validation, document any variations from the validation plan and provide a rationale for them. The variation is provided. To address validation nonconformance, identify the underlying cause and take suitable corrective actions. Validate the system before releasing it for use. You should document any constraints in writing. After verification, an accepted change request should implement system modifications [4].

## Conclusions

A systematic system development life cycle (SDLC), qualification activities throughout the process, and a quality management or assurance system are necessary for a successful CSV. The CSV must define a level of confidence that the system continuously meets requirements and user expectations. Validation of a computer system is required during installation, project operations, and any changes to the software. Validating computer systems can improve data assurance, reduce validation costs and time, increase GMP compliance, and align with 21 CFR Part 11 regulations. CSV framework is essential for safeguarding product quality, adhering to regulatory mandates, and prioritizing patient well-being. Rigorously implementing and maintaining CSV practices is pivotal to achieving these objectives. FDA regulations require computer software validation to ensure product quality, safety, identity, and efficacy, which has an impact on the law. Therefore, we must confirm the system according to quality standards and approved methods to ensure the user's data integrity, traceability, and responsibility. Developing comprehensive CSV strategies that address cybersecurity risks, conducting research on human-computer interaction to optimize system usability and reduce errors, expanding the scope of CSV practices, and enhancing CSV efficiency and effectiveness make improvements in CSV practices.
